# Supplementary material for: Glutamate Supplementation Regulates Nitrogen Metabolism in the Colon and Liver of Weaned Rats Fed a Low-Protein Diet
Source: Nutrients. 2025 Apr 26;17(9):1465. doi: 10.3390/nu17091465 (PMC12073364; doi:10.3390/nu17091465)
Supplement: Supplementary file 1 [file nutrients-17-01465-s001.zip › nutrients-3548397-supplementary.pdf]

nutrients

MDPI

Article

Glutamate Supplementation Regulates Nitrogen Metabolism in the Colon and Liver of Weaned Rats Fed a Low-Protein Diet

Da Jiang, Jing Zhang, Yun Ji, Zhaolai Dai, Ying Yang and Zhenlong Wu *

State Key Laboratory of Animal Nutrition and Feed Science, China Agricultural University, Beijing 100193, China; jayd1994@163.com (D.J.); s20233040807@cau.edu.cn (J.Z.); jean500@163.com (Y.J.); daizhaolai@cau.edu.cn (Z.D.); cauvet@163.com (Y.Y.)

* Correspondence: wuzhenlong@cau.edu.cn; Tel.: +86-10-6273-1003

Table S1. Dietary amino acid composition (Dry matter basis, %).

Items	Calculated Nutrient Level (%)			Analyzed Nutrient Level (%)		
	NCP	LCP + Ala	LCP + Glu	NCP	LCP + Ala	LCP + Glu
Essential amino acids						
Arginine	0.68	0.68	0.68	0.69	0.69	0.68
Histidine	0.56	0.56	0.56	0.55	0.55	0.56
Isoleucine	0.98	0.98	0.98	1.03	1.04	0.99
Leucine	1.76	1.76	1.76	1.74	1.76	1.77
Lysine	1.50	1.50	1.50	1.46	1.45	1.46
Methionine	0.53	0.53	0.53	0.54	0.55	0.55
Phenylalanine	0.97	0.97	0.97	0.93	0.94	0.90
Threonine	0.82	0.82	0.82	0.77	0.81	0.80
Tryptophan	0.24	0.24	0.24	0.27	0.28	0.27
Valine	1.21	1.21	1.21	1.19	1.19	1.19
Nonessential amino acids						
Alanine	0.55	1.52	0.28	0.53	1.55	0.29
Aspartic acid	1.29	0.64	0.64	1.31	0.67	0.68
Cystine	0.38	0.04	0.04	0.36	0.03	0.03
Glutamate	4.12	2.06	4.12	4.14	2.08	4.12
Glycine	0.37	0.19	0.19	0.39	0.16	0.17
Proline	2.16	1.08	1.08	2.20	1.11	1.02
Serine	1.02	0.51	0.51	1.04	0.51	0.53
Tyrosine	1.01	0.51	0.51	1.05	0.54	0.53
EAA + NEAA	20.15	15.80	16.62	20.18	15.91	16.54

Table S2. Sequences of primers used for quantitative real-time PCR.

Genes	Primer Sequences (5′–3′)	GenBank Accession No.	Product (bp)
ATP1A1	F: TCTTGGCTTATGGCATCC<br>R: GGATGCCATAAGCCAAGA	NM_012504.1	288
ATP1A2	F: TTTAGCAATCTCCCTTTAGA<br>R: TCTAAAGGGAGATTGCTAAA	NM_012505.2	198
ATP1A4	F: CTTATTGGCATCATTGTGG<br>R: CCACAATGATGCCAATAAG	NM_022848.3	287
EAAT1	F: GGTATGCACCTCTGGGCA<br>R: TGCCCAGAGGTGCATACC	NM_019225.2	125
EAAT3	F: TATCTTCTGCCTCGTCTTTG<br>R: CAAAGACGAGGCAGAAGATA	NM_013032.3	137
EAAT4	F: CGAGTGGTAACAAGGACGAT<br>R: ATCGTCCTTGTTACCACTCG	NM_032065.2	109
xCT	F: TATCTTCTGCCTCGTCTTTG<br>R: CAAAGACGAGGCAGAAGATA	NM_013032.3	137
4F2hc	F: GGGTGTCTACTGGCAACA<br>R: TGTTGCCAGTAGACACCC	NM_053442.1	210
y^{+}LAT2	F: TCCATAAGAGTGACGCAG<br>R: CTGCGTCACTCTTATGGA	NM_001107424.1	200
$ATB^{0,+}$	F: TCCCTTACCTGACCTACA<br>R: TGTAGGTCAGGTAAGGGA	NM_001037544.2	255
Cat-1	F: AGTGAGTCCCAGACAGGC<br>R: GCCTGTCTGGGACTCACT	NM_001399982.1	255
SNAT9	F: ACCACCATTATCCAAAGA<br>R: TCTTGGATAATGGTGGT	NM_001258286.1	105
B^{0}AT1	F: TCCCTTACCTGACCTACA<br>R: TGTAGGTCAGGTAAGGGA	NM_001037544.2	255
SNAT2	F: ACTTCACCCGATGCTTTC<br>R: GAAAGCATCGGGTGAAGT	NM_181090.3	165
ASCT2	F: GCTCATCCGATTCTTCAAC<br>R: GTTGAAGAATCGGATGAGC	NM_175758.3	255
mGluR1	F: CACATCCTGCTACCTCCA<br>R: TGGAGGTAGCAGGATGTG	NM_017011.2	235
T1R1	F: GGAAACAGAAGAATGGGCA<br>R: TGCCCATTCTTCTGTTCC	NM_053305.1	236
CaSR	F: TGTGCGGCGTAACATCAC<br>R: GTGATGTTACGCCGCACA	NM_001309638.1	211
GAPDH	F: ATGTCGTGGAGTCTACTGGC<br>R: GCCAGTAGACTCCACGACAT	NM_017008.4	140

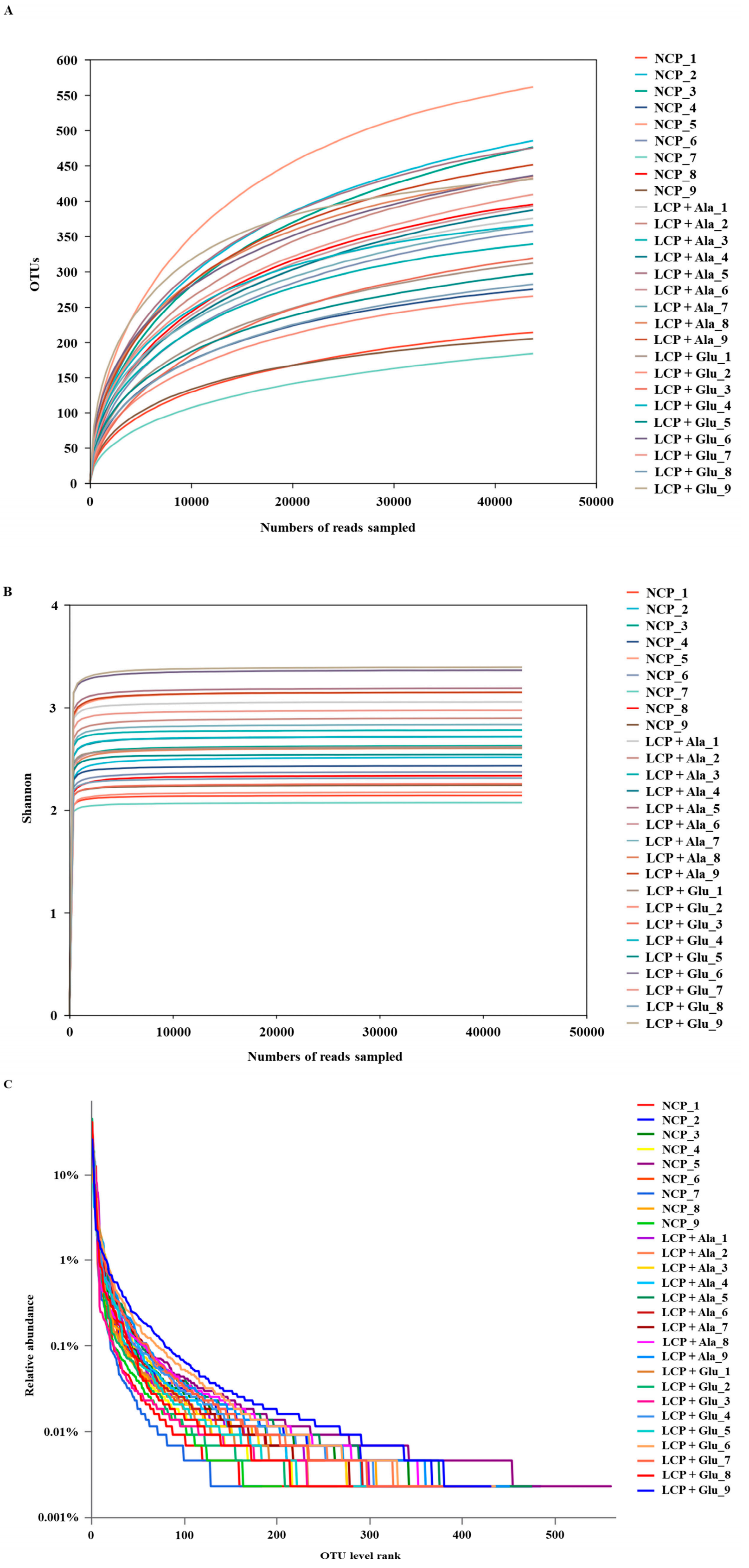

Figure S1. Evaluation of data from Illumina Miseq sequencing. Rarefaction (A), shannon (B), and rank-abundance (C) obtained from each sample.
